# Young-Adult Polycystic Kidney Disease is Associated with Major Cardiovascular Complications

**DOI:** 10.3390/ijerph15050903

**Published:** 2018-05-03

**Authors:** Ya-Wen Chuang, Tung-Min Yu, Shih-Ting Huang, Kuo-Ting Sun, Ying-Chih Lo, Pin-Kuei Fu, Bor-Jen Lee, Cheng-Hsu Chen, Cheng-Li Lin, Chia-Hung Kao

**Affiliations:** 1 Division of Nephrology, Taichung Veterans General Hospital, Taichung 40447, Taiwan; colaladr@yahoo.com.tw (Y.-W.C.); yu5523@gmail.com (T.-M.Y.); kitheroborn@hotmail.com (S.-T.H.); neversee@ms41.hinet.net (Y.-C.L.); cschen920@yahoo.com (C.-H.C.); 2 Graduate Institute of Clinical Medical Science and School of Medicine, College of Medicine, China Medical University, Taichung 40447, Taiwan; duke111053@hotmail.com; 3 Pediatric Dentistry of Dental Department, China Medical University Hospital, Taichung 40447, Taiwan; 4 Department of Critical Care, Taichung Veterans General Hospital, Taichung 40447, Taiwan; yetquen@gmail.com (P.-K.F.); borjen@mail.vghtc.gov.tw (B.-J.L.); 5 Management Office for Health Data, China Medical University Hospital, Taichung 40447, Taiwan; orangechengli@gmail.com; 6 College of Medicine, China Medical University, Taichung 40447, Taiwan; 7 Department of Nuclear Medicine and PET Center, China Medical University Hospital, Taichung 40447, Taiwan; 8 Department of Bioinformatics and Medical Engineering, Asia University, Taichung 40447, Taiwan

**Keywords:** polycystic kidney disease, acute coronary syndrome (ACS), stroke, congestive heart failure (CHF), survival

## Abstract

*Background:* Patients with polycystic kidney disease (PKD) might have a risk of cardiovascular diseases because several cardiovascular risk factors are occasionally associated with PKD patients. Data on the association between PKD and the risk of cardiovascular events, including acute coronary syndrome (ACS), stroke, and congestive heart failure (CHF), are scant. *Methods:* Patients aged ≥20 years who were newly diagnosed with PKD (International Classification of Diseases, Ninth Revision, Clinical Modification codes 753.12 and 753.13) between 2000 and 2011 were selected as a PKD cohort (N = 5157). The association between PKD and cardiovascular events was analyzed. *Results:* We randomly selected a comparison cohort of people without PKD, who were frequency-matched by sex, age, and index date of diagnosis. At the end of 2011, the PKD cohort had a 1.40-fold greater incidence of ACS compared with the comparison cohort (8.59 vs. 6.17 per 1000 person-years), in addition to a 1.40-fold greater incidence of stroke, a 1.49-fold greater incidence of CHF, and a 1.64-fold greater incidence of mortality. *Conclusions:* This retrospective cohort study shows that patients with PKD have an increased risk of cardiovascular events including ACS, stroke, and CHF as well as mortality, particularly in younger patients. Early identification is necessary to attenuate the risk of cardiovascular complications in patients with PKD.

## 1. Background

Polycystic kidney disease (PKD), a multisystem disorder of ciliary proteins, is occasionally complicated by extra-renal manifestations [1]. The most prevalent PKD in adulthood is autosomal dominant PKD (ADPKD), which is also the most common detrimental monogenetic disorder [1,2]. Although large interfamilial and intrafamilial variations exist in patients with PKD, mutations of the PKD1 (chromosome region 16p13.3, approximately 85% of cases) and PKD2 (4q21, approximately 15% of cases) genes account for most PKD cases [2]. 

Previous study suggested that polycystins are associated with the vascular abnormality of blood vessels [3].

Polycystin 1 (PC1) and polycystin 2 (PC2), the defect proteins in ADPKD, are found in both vascular smooth muscle cells (VSMC) and endothelial cells that are involved in all major vessels including the aorta and intracranial arterials [4,5,6]. The interactions between PC1/PC2 and VSMCs are complex. Previous data indicated that the dysregulation of Ca^2+^ homeostasis, which resulted from the mutation of PC1/PC2, may lead to an increase in VSMC proliferation and apoptosis resembling the P*kd*-mutant phenotype [7].

Moreover, several lines of studies suggested that the mTOR signaling pathway is involved with patho-physiological changes of VSMCs including proliferation, differentiation, and migration with increased extracellular matrix protein synthesis [8]. 

VSMCs after injury are frequently prone to abnormal proliferation and matrix synthesis which eventually contributes to intimal hyperplasia and lumen narrowing in vessels [9]. 

The mTOR pathway inhibitor is shown to be effective in the attenuation of intimal hyperplasia in an animal model and clinical trials [10,11]. 

There is overwhelming evidence for the hyperactivity of mTOR signaling in PKD [12,13]. Edelstein et al. firstly found that mTOR could involve the progression of cyst changes in the Han: SPRD rat model of PKD [14]. In addition, Shillingford et al. further demonstrated a substantial role of mTOR contributing to the pathogenesis of human PKD and suggested that patients with PKD are likely to be associated with an aberrant activity of mTOR [15].

Accordingly, it is reasonable to suppose that PKD is associated with a higher risk of vascular complications because of the hyperactivity of mTOR resulting from a PC1/2 deficiency.

In patients with PKD, the underlying defect in the connective tissue matrix has been associated with various extrarenal manifestations including intracranial aneurysms, hepatic cysts, diverticulosis, spontaneous coronary artery dissection (SCAD), atrial fibrillation [16], and other cardiovascular abnormalities [17].

However, data regarding the relationship between PKD and the risk of cardiovascular events such as coronary artery disease and cerebral vascular disease remain limited, particularly for acute coronary syndrome (ACS) characterized by unstable angina with a non-ST-segment elevation myocardial infarction and ST-segment elevation myocardial infarction, which is a life-threatening condition [18,19].

In the current study, we used the large claims database of the Taiwan National Health Insurance (NHI) program to conduct a nationwide cohort study to investigate the risk of cardiovascular events, including ACS and stroke, in patients with PKD.

## 2. Materials and Methods

### 2.1. Data Source

The NHI program covers more than 99% of the population of Taiwan (http://www.nhi.gov.tw/english/index.aspx). The Bureau of National Health Insurance authorized the Taiwan National Health Research Institutes to manage the electronic medical records of patients enrolled in this program and establish the National Health Insurance Research Database (NHIRD) for research purposes. Information available in the database includes the demographic status of insurants; medical diagnoses, treatment procedures, and drug prescriptions for inpatient and outpatient services; and healthcare facilities providing care services to patients. For this study, we used a subset of the NHIRD containing health care data including files of inpatients claims, and the Registry of Beneficiaries. To ensure the privacy of personal information, unique encrypted identification numbers are used for all patients. All claimed data sets can be linked using these anonymous identification numbers. All diagnoses were defined using the International Classification of Diseases, Ninth Revision, Clinical Modification (ICD-9-CM). 

### 2.2. Data Availability Statement

The dataset used in this study is held by the Taiwan Ministry of Health and Welfare (MOHW). The Ministry of Health and Welfare must approve our application to access this data. Any researcher interested in accessing this dataset can submit an application form to the Ministry of Health and Welfare requesting access. Please contact the staff of MOHW (Email: stcarolwu@mohw.gov.tw) for further assistance. Taiwan Ministry of Health and Welfare Address: No. 488, Sec. 6, Zhongxiao E. Rd., Nangang Dist., Taipei City 115, Taiwan (R.O.C.). Phone: +886-2-8590-6848. All relevant data are within the paper.

### 2.3. Ethics Statement

The NHIRD encrypts patient personal information to protect privacy and provides researchers with anonymous identification numbers associated with relevant claims information, including sex, date of birth, medical services received, and prescriptions. Therefore, patient consent is not required to access the NHIRD. This study was approved to fulfill the condition for exemption by the Institutional Review Board (IRB) of China Medical University (CMUH104-REC2-115-CR2). The IRB also specifically waived the consent requirement.

### 2.4. Study Patients

**Figure 1:**
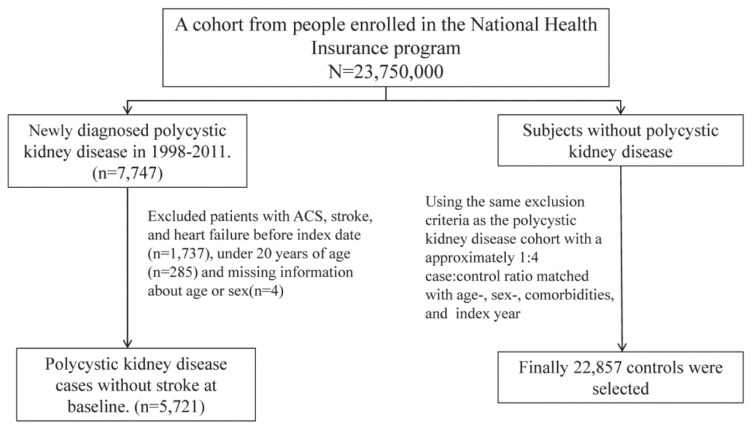
Shows the selection process of the participants in the 2 study cohorts.

Figure 1 shows the selection process of the participants in the 2 study cohorts. From the inpatient database, we chronologically identified 5721 patients aged ≥20 years who were newly diagnosed with PKD (ICD-9-CM codes 753.12 and 753.13) between 2000 and 2011. The diagnosis date was designated as the “start date” of the follow-up period (in years). For each PKD patient, we randomly selected 4 controls frequency-matched by sex, age, start date, and comorbidities of hypertension (ICD-9-CM codes 401–405), diabetes (ICD-9-CM code 250), chronic obstructive pulmonary disease (COPD; ICD-9-CM codes 491, 492, and 496), chronic kidney disease (CKD; ICD-9-CM code 585), hyperlipidemia (ICD-9-CM code 272), and end-stage renal disease (ESRD; ICD-9-CM code 585 with catastrophic certification). We gave the controls (non-PKD cohort) the same start year as cases (PKD cohort) and then randomly assigned months and days to the controls as the start date of the controls. The study populations in both cohorts were followed from the start dates until the development of ACS or other events of interest, until they were censored for loss to follow-up or withdrawal from the NHI program, or until the end of 2011. The primary goal of this study was to compare the risk of 3 outcomes and mortality between patients with and without PKD: (1) ACS (ICD-9-CM codes 410, 411.1, and 411.8) including unstable angina (ICD-9-CM code 411.1), NSTEMI (ICD-9-CM codes 410.70–410.72), and STEMI (ICD-9-CM codes 410.0–410.9, except for codes 410.70–710.72); (2) stroke (ICD-9-CM codes 430–438) including hemorrhagic stroke (ICD-9-CM codes 430–432) and ischemic stroke (ICD-9-CM codes 433–438); and (3) heart failure (ICD-9-CM code 428) as well as patient death. Patients identified with these disorders were excluded at the baseline.

### 2.5. Statistical Analysis

Distributions of sex, age (20–49, 50–64, and ≥65 years), and comorbidities (yes and no) were compared between the cohorts with and without PKD. We used the Chi-squared test to examine categorical variables and the Wilcoxon rank-sum test to examine the skewed age distributions between the groups. For events significantly associated with PKD, we used the Kaplan–Meier method to calculate and plot the cumulative incidences of such events and examined the differences between the cohorts by using the log-rank test. We also calculated the overall incidence rates of each study event in both cohorts. Because mortality was a critical factor affecting the estimation of ACS, stroke, and heart failure risk, we considered the event of death as a competing event to estimate subhazard ratios (SHRs) and 95% confidence intervals (CIs) by using the standard univariable and multivariable Cox proportional hazards regression models [20]. Cox regression models were used to calculate the crude hazard ratios (HRs) and 95% CIs of PKD-related mortality in the PKD and non-PKD cohorts. We also used events with a significant crude SHR (cSHR) or crude HR to calculate the relative adjusted SHR (aSHR) or adjusted HR (aHR) of the events associated with PKD through multivariable Cox regression analysis after controlling the baseline characteristics. The joint effect between PKD and the comorbidities was also measured for outcome events that were significantly associated with PKD. SAS 9.3 statistical software (SAS Institute, Inc., Cary, NC, USA) was used for all statistical analyses. The 2-sided significance level was set at 0.05.

## 3. Results

**Figure 2:**
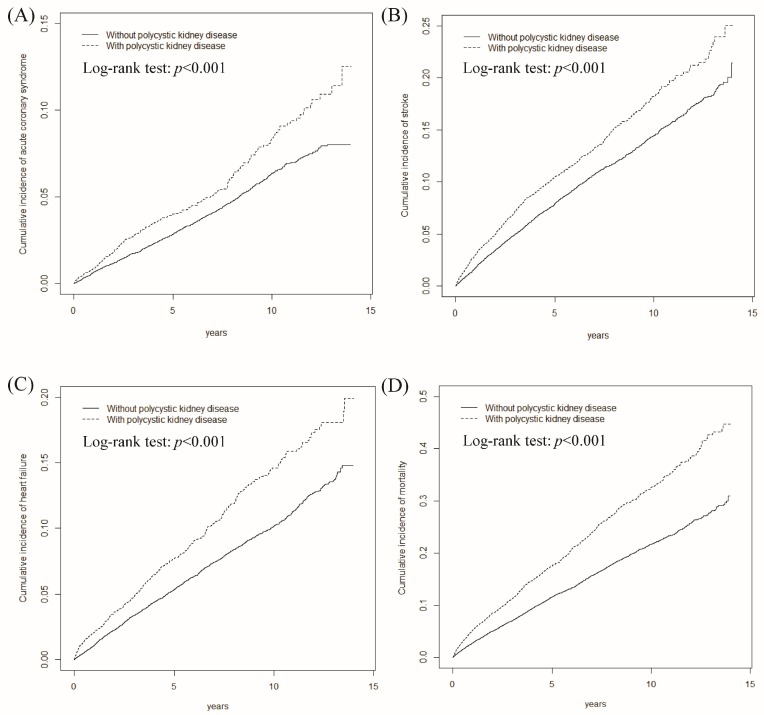
Cumulative incidence of acute coronary syndrome (**A**); stroke (**B**); congestive heart failure (**C**); and mortality (**D**) for polycystic kidney disease cohort (dashed line) and comparison cohort (solid line).

Table 1 presents the baseline data of the PKD and comparison cohorts; the baseline characteristics were similar in both cohorts. As shown in Figure 2, the cumulative incidence rates of ACS, stroke, congestive heart failure (CHF), and mortality were all greater in the PKD cohort than in the non-PKD cohort.

Table 2 shows the overall incidence rates of the 3 events between the PKD and non-PKD cohorts as well as the cSHRs of the events in these cohorts. The PKD patients were at a higher risk of all events than the comparisons. Overall, comparing the PKD and non-PKD cohorts revealed that the PKD cohort had a higher incidence of ACS (8.59 vs. 6.17 per 1000 person-years), unstable angina (1.98 vs. 1.26 per 1000 person-years), stroke (21.5 vs. 16.1 per 1000 person-years), hemorrhagic stroke (6.16 vs. 2.97 per 1000 person-years), ischemic stroke (15.3 vs. 13.2 per 1000 person-years), and CHF (16.3 vs. 11.1 per 1000 person-years), corresponding to cSHR values of 1.40 (95% CI = 1.22–1.61), 1.41 (95% CI = 1.05–1.89), 1.40 (95% CI = 1.28–1.53), 2.12 (95% CI = 1.78–2.53), 1.20 (95% CI = 1.08–1.33), and 1.49 (95% CI = 1.34–1.65), respectively. The sex-specific PKD-to-non-PKD cohort relative risk of ACS was significant for men (cSHR = 1.47, 95% CI = 1.24–1.74). Furthermore, the age-specific PKD-to-non-PKD relative risk of ACS was high for all age groups. Notably, in the group aged 20–49 year-old, a 1.61-fold increased risk of ACS (95% CI = 1.18, 2.21), 1.65-fold in stroke (95% CI = 1.32, 2.07), and 1.82-fold in CHF (95% CI = 1.37, 2.42) was noted.

PKD patients with comorbidities were associated with significantly higher risks of ACS than the non-PKD patients. The PKD cohort was 1.40-fold more likely to develop stroke (95% CI = 1.28–1.53), 2.12-fold more likely to develop hemorrhagic stroke (95% CI = 1.78–2.53), and 1.20-fold more likely to develop ischemic stroke (95% CI = 1.08–1.33) compared with the non-PKD cohort. We compared the risks of stroke and CHF between the PKD and non-PKD cohorts in terms of several variables including sex, age, and the presence or absence of comorbidities. In all stratifications, the risks of stroke and CHF in the PKD cohort were higher than those in the non-PKD cohort, except for patients without comorbidities.

Table 3 shows a significantly high risk of mortality in patients with PKD compared to that in patients without PKD which reached a 1.64-fold increase (95% CI = 1.53–1.75). In stratification with sex and age shows that the sex-specific PKD-to-non-PKD cohort relative risk of mortality was significant for men (cHR = 1.60, 95% CI = 1.47–1.73) as well as that in women (cHR = 1.75, 95% CI = 1.57–1.97). Furthermore, the age-specific PKD-to-non-PKD relative risk of mortality was high for all age groups. Notably, in the group aged 20–49 year-old, a 1.84-fold increased risk of mortality (95% CI = 1.55, 2.18). 

Table 4 presents the calculated cSHRs and aSHRs of the 3 PKD-related events and cHRs and aHRs of the mortality after adjustment for sex, age, and baseline comorbidities. Men were at higher risks of ACS, stroke and mortality than women. The hazard of heart failure was higher for patients aged 50–64 years than for those aged 20–49 years. Hypertension, diabetes, CKD, and ESRD were associated with increased risks of ACS, stroke, CHF, and mortality.

## 4. Discussion

In this large population-based cohort study with a long observation period of 27,053 person-years, we noted that PKD patients had a significantly higher risk of ACS as well as CHF when compared with non-PKD patients after adjustment for age, sex, and the associated cardiovascular risk factors included hypertension, diabetes, hyperlipidemia, COPD, and CKD. We also found greater risks of stroke and mortality for the PKD cohort than for the non-PKD cohort. Interestingly, our findings disclosed a significantly increased risk of cardiovascular events including ACS, stroke, and CHF in young patients with PKD, which has never been reported previously for such a relatively young age group.

Cardiovascular complications have surpassed infections among the most common causes of morbidity and mortality in patients with PKD [18,19]. Some cardiovascular risk factors have been suggested to be closely associated with PKD. Hypertension and left ventricular hypertrophy are commonly observed in patients with PKD [19,21]. Early vascular changes have been reported in young patients with normal blood pressure, and patients with PKD are considered to have a higher prevalence of coronary aneurysm (CA) and cardiac valvular abnormalities than the general population does [19]. Nevertheless, diagnosing SCAD is extremely challenging. Even in patients with a strong clinical suspicion, SCAD is difficult to diagnose accurately by using coronary angiography [22]. Previous studies on SCAD associated with PKD are limited to a few case reports and a study with a small sample size [23,24,25]. 

In the present study, after adjusting for associated risk factors including hypertension, diabetes, hyperlipidemia, and chronic kidney disease, multivariate analysis revealed a relatively high hazard of ACS as well as stroke in patients with PKD compared with non-PKD patients. Notably, the age-specific risk of ACS was significantly higher for those who were younger, aged 20–49 years (HR = 1.61, 95% CI = 1.18–2.21), and additionally a 1.82-fold increased risk of CHF was observed in young people with PKD. In addition, the trend is consistent in that a 1.65-fold increased risk of stroke was observed. 

More recently, a study on PKD patients indicated that a high prevalence of young-onset hypertension was observed in children with PKD and that may partly support the findings in the present study.

In terms of stratification without co-morbidity, our results showed that the risk of CVD was comparable between patients with and without PKD. The findings may suggest that the associated cardiovascular co-morbidity may contribute more greatly in patients once they have PKD. 

Taken together, our findings indicate a higher risk of cardiovascular events in younger patients with PKD that was never previously known. 

Previous studies have shown that polycystins are constitutional in normal adult smooth muscle cells [5,6]. Polycystins may play an essential role in maintaining the vascular integrity of the myoelastic structure of the arterial wall [26]. An animal study showed that the null mutation of the PKD1 or PKD2 gene could diffuse vascular rupture and hemorrhage in the vessel tissue, suggesting that polycystins have a role in maintaining vascular integrity [5,27]. Hence, polycystin deficiency is suggested to be directly attributed to the various phenotypes of vascular abnormality in patients with ADPKD, which are independent of the effect of hypertension [6]. 

Moreover, accumulating evidence indicates that PKD is likely associated with aberrant mTOR activity. Previous study demonstrated that the cytoplasmic tail of PC1 could inhibit mTOR and the loss function of PC1 in ADPKD would eventually lead to a remarkable hyperactivity of mTOR [13,15].

We previously demonstrated that PKD is significantly associated with the risk of cancer, within which mTOR may play an important role [28]. Whether mTOR has a similar role within it needs further elucidation.

Our study results may provide clinical evidence relevant to previous biological evidence.

Previous study has suggested that a rennin-angiotensin blockade was likely associated with a favorable outcome in PKD patients. Therefore, in terms of cardiovascular complications, it should be firstly considered in PKD patients. Certain limitations of the present study should be considered. First, some data on tobacco smoking and kidney function as glomerular filtration rate are relevant to the risk of ACS but are not provided in the NHIRD. Therefore, we included the confounder of COPD, chronic kidney disease, and end stage renal disease (ESRD) in the multivariable analysis to correct any possible bias. Second, we could not obtain details regarding CA and SCAD. The clinical diagnosis of SCAD is challenging and relies completely on visualizing a radiolucent intimal “flap” in coronary angiography [22,29]. In practice, delineating the coronary wall through ordinary cardiac angiography is difficult, limiting its diagnostic accuracy [22,29,30]. Nevertheless, the diagnoses of ACS and AMI in the study were not biased by this condition. Third, we did not consider the effects of medications including antihypertensive, antiplatelet, and antidiabetic drugs. This may have led to an underestimation of the risk of ACS in the study. Finally, we could not analyze biological data obtained through genetic analysis including the mutations of the PKD1 and PKD2 genes. We could not examine the individual risks of vascular phenotypes in the study cohort, although this is not necessary for the contemporary diagnosis of PKD or ADPKD.

## 5. Conclusions

Our study reports for the first time that patients with PKD are at increased risk of ACS as well as other cardiovascular events including stroke, CHF, and mortality.

Most important of all, our findings show that young adult PKD is significantly associated with a higher risk of cardiovascular disease when compared with non-PKD patients. The relatively high risk of cardiovascular events such as stroke and heart failure, as well as mortality, should not be ignored in young patients with PKD, and early recognition of the cardiovascular risk in such patients is required. 

## Reference

## Figures and Tables

**Table 1:** Characteristics of patients between patients with and without polycystic kidney disease (PKD).

Age, Year	Polycystic Kidney Disease				*p*-Value
	Yes		No		
	(N = 5721)		(N = 22,857)		
	N	%	N	%	
					0.99
20–49	2080	36.4	8308	36.4	
50–64	1676	29.3	6695	29.3	
≥65	1965	34.4	7854	34.4	
Median (IQR) ^#^	56.0	(45.1–70.3)	56.1	(45.1–70.5)	0.76
Gender					0.98
Female	2250	44.6	10,192	44.6	
Male	3171	55.4	12,665	55.4	
Comorbidity					
Hypertension	2504	43.8	10,006	43.8	0.99
Diabetes	610	10.7	2432	10.6	0.96
Hyperlipidemia	236	4.13	933	4.08	0.88
COPD	327	5.72	1293	5.66	0.86
Chronic kidney disease	1234	21.6	4915	21.5	0.91
ESRD	564	9.86	2239	9.80	0.89

Chi-square test; ^#^ Wilcoxon signed rank test. IQR: interquartile range. COPD: chronic obstructive pulmonary disease. ESRD: end-stage renal disease.

**Table 2:** Incidence and hazard ratio of outcome between patients with and without polycystic kidney disease.

Variable	Polycystic Kidney Disease								Crude SHR ^†^ (95% CI)
	Yes				No				
	N	Event	PY	Rate ^#^	N	Event	PY	Rate ^#^	
ACS									
All	5721	252	29,323	8.59	22,857	790	128,080	6.17	1.40(1.22, 1.61)
Unstable angina		58		1.98		161		1.26	1.41(1.05, 1.89)
NSTMI		21		0.72		77		0.60	0.94(0.59, 1.50)
STMI		69		2.35		238		1.86	1.14(0.88, 1.49)
Gender									
Female	2550	72	14,002	5.14	10,192	245	59,827	4.10	1.26(0.98, 1.64)
Male	3171	180	15,322	11.8	12,665	545	68,253	7.99	1.47(1.24, 1.74)
Age, year									
20–49	2080	54	12,584	4.29	8308	131	51,120	2.56	1.61(1.18, 2.21)
50–64	1676	84	8865	9.48	6695	216	38,289	5.64	1.67(1.30, 2.15)
≥65	1965	114	7874	14.5	7854	443	38,671	11.5	1.28(1.04, 1.57)
Comorbidity ^§^									
No	37	2	268	7.47	148	7	1101	6.36	1.11(0.23, 5.24)
Yes	5684	250	29,055	8.60	22,709	783	126,978	6.17	1.40(1.22, 1.61)
Stroke									
All	5721	609	28,395	21.5	22,857	2002	124,216	16.1	1.40(1.28, 1.53)
Hemorrhagic stroke		175	28,396	6.16		369	124,216	2.97	2.12(1.78, 2.53)
Ischemic stroke		434	28,396	15.3		1633	124,216	13.2	1.20(1.08, 1.33)
Gender									
Female	2550	237	13,533	17.5	10,192	677	58,390	11.6	1.62(1.40, 1.88)
Male	3171	372	14,863	25.0	12,665	1325	65,826	20.1	1.29(1.15, 1.45)
Age, year									
20–49	2080	108	12,419	8.70	8308	265	50,742	5.22	1.65(1.32, 2.07)
50–64	1676	176	8628	20.4	6695	537	37,243	14.4	1.41(1.19, 1.67)
≥65	1965	325	7349	44.2	7854	1200	36,231	33.1	1.35(1.19, 1.52)
Comorbidity ^§^									
No	37	4	254	15.7	148	17	1060	16.0	0.98(0.33, 2.89)
Yes	5684	605	28,142	21.5	22,709	1985	123,156	16.1	1.41(1.28, 1.54)
Congestive heart failure									
All	5721	471	28,987	16.3	22,857	1410	126,905	11.1	1.49(1.34, 1.65)
Gender									
Female	2550	171	13,806	12.4	10,192	531	59,153	8.98	1.43(1.21, 1.69)
Male	3171	300	15,181	19.8	12,665	879	67,751	13.0	1.52(1.34, 1.73)
Age, year									
20–49	2080	69	12,585	5.48	8308	150	51,123	2.93	1.82(1.37, 2.42)
50–64	1676	116	8866	13.1	6695	336	37,950	8.85	1.46(1.18, 1.80)
≥65	1965	286	7537	38.0	7854	924	37,832	24.4	1.57(1.38, 1.80)
Comorbidity ^§^									
No	37	0	276	0.00	148	7	1107	6.32	-
Yes	5684	471	28,711	16.4	22,709	1403	125,798	11.2	1.50(1.35, 1.66)

ACS, acute coronary syndrome. PY, person-years; Rate ^#^, incidence rate per 1000 person-years. Crude SHR ^†^, relative hazard ratio. Comorbidity ^§^: Patients with any one of the comorbidities (including hypertension, diabetes, hyperlipidemia, COPD, chronic kidney disease and ESRD) were classified as the comorbidity group. NSTMI: non-ST elevation myocardial infarction. STMI: ST elevation myocardial infarction.

**Table 3:** Incidence and hazard ratio of mortality between patients with and without polycystic kidney disease.

Variable	Polycystic Kidney Disease								Crude HR ^†^ (95% CI)
	Yes				No				
	N	Event	PY	Rate ^#^	N	Event	PY	Rate ^#^	
All	5721	1217	30,045	40.5	22,857	3218	130,270	24.7	1.64(1.53, 1.75)
Gender									
Female	2550	416	14,224	29.3	10,192	1012	60,548	16.7	1.75(1.57, 1.97)
Male	3171	801	15,822	50.6	12,665	2206	69,721	31.6	1.60(1.47, 1.73)
Age, year									
20–49	2080	191	12,778	15.0	8308	421	51,648	8.15	1.84(1.55, 2.18)
50–64	1676	347	9154	37.9	6695	875	38,931	22.5	1.69(1.50, 1.92)
≥65	1965	679	8113	83.7	7854	1922	39,690	48.4	1.73(1.59, 1.89)
Comorbidity ^§^									
No	37	7	276	25.3	148	13	1122	11.6	2.14(0.86, 5.38)
Yes	5684	1210	29,769	40.7	22,709	3205	129,148	24.8	1.64(1.53, 1.75)

PY, person-years; Rate ^#^, incidence rate per 1000 person-years. Crude HR ^†^, relative hazard ratio. Comorbidity ^§^: Patients with any one of the comorbidities (including hypertension, diabetes, hyperlipidemia, COPD, chronic kidney disease and ESRD) were classified as the comorbidity group.

**Table 4:** Hazard ratios of outcome in association with gender, age and comorbidities in univariable and multivariable competing risk models.

Variable	ACS		Stroke		Congestive Heart Failure		Mortality	
	Crude SHR ^†^ (95% CI)	Adjusted SHR ^‡^ (95% CI)	Crude SHR ^†^ (95% CI)	Adjusted SHR ^‡^ (95% CI)	Crude SHR ^†^ (95% CI)	Adjusted SHR ^‡^ (95% CI)	Crude HR ^†^ (95% CI)	Adjusted HR ^‡^ (95% CI)
PKD	1.40(1.22, 1.61)	1.40(1.22, 1.62)	1.40(1.28, 1.53)	1.39(1.27, 1.52)	1.49(1.34, 1.65)	1.51(1.36, 1.68)	1.64(1.53, 1.75)	1.71(1.60, 1.83)
Gender (Men vs. women)	1.58(1.39, 1.79)	1.55(1.36, 1.77)	1.30(1.20, 1.40)	1.27(1.17, 1.38)	1.07(0.98,1.17)	1.03(0.94, 1.13)	1.84(1.73, 1.96)	1.42(1.33, 1.51)
Age, years								
20–49	1(Reference)	1(Reference)	1(Reference)	1(Reference)	1(Reference)	1(Reference)	1(Reference)	1(Reference)
50–64	1.37(1.03, 1.82)	1.19(0.89, 1.59)	1.28(1.04, 1.57)	1.04(0.84, 1.28)	1.79(1.40, 2.29)	1.45(1.13, 1.84)	2.69(2.44, 2.96)	2.32(2.11, 2.56)
≥65	1.23(0.99, 1.54)	1.08(0.87, 1.35)	1.25(1.07, 1.46)	1.12(0.96, 1.30)	1.31(1.08, 1.58)	1.10(0.91, 1.33)	5.78(5.29, 6.32)	5.16(4.72, 5.65)
Baseline comorbidities (yes vs. no)								
Hypertension	1.96(1.74, 2.21)	1.76(1.55, 1.99)	1.61(1.49, 1.74)	1.51(1.39, 1.63)	1.69(1.54, 1.85)	1.53(1.39, 1.68)	1.69(1.59, 1.79)	1.21(1.14, 1.28)
Diabetes	2.21(1.90, 2.57)	1.87(1.60, 2.18)	1.79(1.62, 1.98)	1.65(1.48, 1.83)	2.20(1.97, 2.45)	2.00(1.78, 2.24)	2.07(1.92, 2.24)	1.67(1.54, 1.81)
Hyperlipidemia	2.40(1.95, 2.95)	1.87((1.51, 2.32)	1.41(1.20, 1.67)	1.12(0.95, 1.33)	1.69(1.41, 2.02)	1.33(1.10, 1.59)	1.07(0.93, 1.24)	0.88(0.76, 1.02)
COPD	1.37(1.10, 1.70)	1.16(0.93, 1.44)	1.42(1.25, 1.61)	1.30(1.14, 1.48)	1.72(1.50, 1.98)	1.52(1.32, 1.76)	2.76(2.51, 3.03)	1.60(1.45, 1.76)
CKD	2.20(1.94, 2.49)	1.90(1.65, 2.20)	1.73(1.60, 1.89)	1.64(1.49, 1.81)	2.52(2.30, 2.76)	2.37(2.14, 2.63)	3.36(3.17, 3.56)	2.76(2.58, 2.95)
ESRD	2.12(1.78, 2.52)	1.32(1.07, 1.61)	1.52(1.33, 1.73)	1.03(0.89, 1.20)	2.19(1.92, 2.50)	1.29(1.11, 1.50)	2.80(2.60, 3.02)	1.60(1.47, 1.74)

Crude SHR ^†^, relative subhazard ratio; Crude HR ^†^, relative hazard ratio; Adjusted SHR ^‡^ and Adjusted HR ^‡^: multivariable analysis including gender, age, and comorbidities of hypertension, diabetes, hyperlipidemia, COPD, chronic kidney disease (CKD), and ESRD.

## Abbreviations

PKD	polycystic kidney disease
ACS	acute coronary syndrome
CHF	congestive heart failure
CI	confidence interval
VSMC	vascular smooth muscle cells
NHIRD	National Health Insurance Research Database
ICD-9-CM	International Classification of Diseases, Ninth Revision, Clinical Modification
HR	hazard ratio
